# Ablation of PI3Kγ in neurons protects mice from diet-induced obesity MASLD and insulin resistance

**DOI:** 10.1016/j.isci.2024.111562

**Published:** 2024-12-09

**Authors:** Angela Molinaro, Arianna Mazzoli, Andrea Usseglio Gaudi, Amit Chand Gupta, Vagner Ramon Rodrigues Silva, Damien Ramel, Muriel Laffargue, Johan Ruud, Barbara Becattini, Giovanni Solinas

**Affiliations:** 1 The Wallenberg Laboratory, Institute of Medicine University of Gothenburg Sweden, Gothenburg, Sweden; 2 Institute of Metabolic and Cardiovascular Diseases (I2MC), Institut National de la Santé et de la Recherche Médicale (INSERM) U1297, University of Toulouse 3, Toulouse, France; 3 Department of Physiology, Institute of Neuroscience and Physiology, Sahlgrenska Academy, University of Gothenburg, Gothenburg, Sweden

**Keywords:** Molecular biology, Neuroscience

## Abstract

Mice with genetic ablation of PI3Kγ are protected from diet-induced obesity. However, the cell type responsible for PI3Kγ action in obesity remains unknown. We generated mice with conditional deletion of PI3Kγ in neurons using the nestin promoter to drive the expression of the Cre recombinase (PI3Kγ^Nest^ mice) and investigated their metabolic phenotype in a model of diet-induced obesity. On a chow diet, lean PI3Kγ^Nest^ mice display reduced linear growth and a normal metabolic phenotype. PI3Kγ^Nest^ mice were largely protected from diet-induced obesity and liver steatosis and showed improved glucose tolerance and insulin sensitivity. This phenotype was associated with increased phosphorylation of hormone-sensitive lipase (HSL) at protein kinase A (PKA) sites in white fat. It is concluded that PI3Kγ action in diet-induced obesity depends on its activity in neurons controlling adipose tissue lipolysis. Future clinical studies on PI3Kγ inhibitors capable of crossing the brain-blood barrier will reveal the relevance of these findings to humans.

## Highlights

• Loss of PI3Kγ in neurons protects mice from diet-induced obesity
• Mice lacking PI3Kγ in neurons display improved insulin sensitivity
• Mice lacking PI3Kγ in neurons are protected from fatty liver disease
• Loss of PI3Kγ in neurons increases adipose tissue HSL phosphorylation at PKA sites

## Graphical abstract

**Figure:**
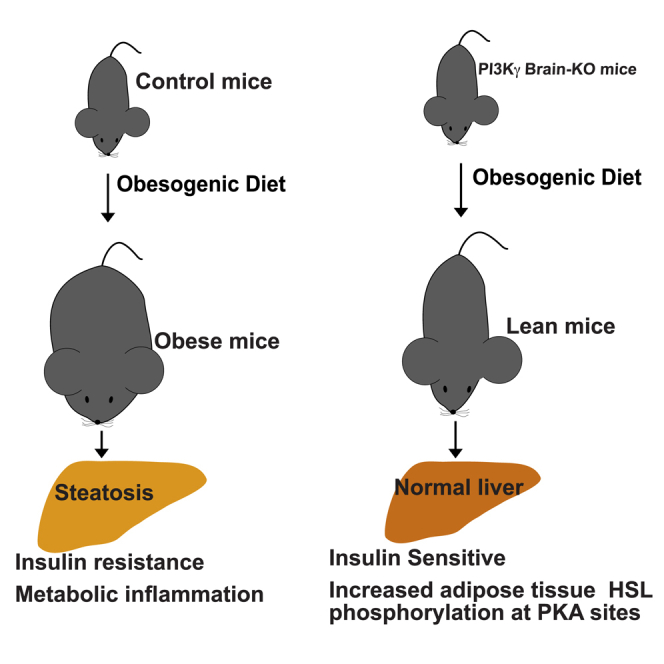

## Introduction

Class-1 phosphoinositide-3 kinases (PI3K) are heterodimeric proteins composed of one of four catalytic subunits, PI3Kα, PI3Kβ, PI3Kγ, and PI3Kδ, in a complex with a regulatory protein.^1^ The combinatorial nature of PI3K defines the functional redundancies and specificities of different isoforms in the transduction of signals controlling cell metabolism and growth.^2^^,^^3^^,^^4^

In cancer, several common driver mutations result in constitutive PI3K activation.^5^ However, due to the essential role of PI3K activity in insulin signal transduction, pan-PI3K inhibition leads to hyperglycemia and marked hyperinsulinemia.^6^ The latter induces PI3K activity in insulin-sensitive tumors, causing resistance to PI3K-targeted therapies.^7^ Understanding the role of different PI3K in metabolic homeostasis is, therefore, key to the development of effective PI3K-targeted therapies.

Studies from different laboratories also demonstrate that PI3K activity controls adiposity in rodents, monkeys, and humans.^8^^,^^9^^,^^10^^,^^11^^,^^12^^,^^13^^,^^14^^,^^15^^,^^16^^,^^17^^,^^18^ However, effective PI3K-targeted therapy for obesity should separate the beneficial effect of PI3K inhibition on reduced adiposity from its deleterious effects on blood glucose and hyperinsulinemia. This might be achieved by selective inhibition of PI3Kγ.^8^^,^^9^^,^^12^^,^^13^^,^^14^ Mice lacking PI3Kγ do not develop hyperinsulinemia or hyperglycemia and are protected from diet-induced obesity, insulin resistance, and metabolic dysfunction associated steatotic liver disease (MASLD),^8^^,^^9^^,^^13^^,^^14^ and obesity-promotion of hepatocellular carcinoma.^19^ Studies combining PI3Kα tissue-specific conditional knockout mice with the administration of a panel of isoform-selective inhibitors indicate that PI3Kγ does not play a significant role in insulin signaling in hepatocytes and adipocytes.^18^^,^^20^ These results explain why mice with PI3Kγ ablation are not predisposed to insulin resistance and indicate that the PI3Kγ action in promoting adiposity is not due to its activity in the adipocyte or the hepatocyte. Although the loss of PI3Kγ protects mice from diet-induced obesity,^8^^,^^9^^,^^13^^,^^14^ it does not reduce adiposity in leptin-deficient mice or mice lacking a functional leptin receptor.^9^^,^^13^^,^^21^ Because selective deletion of leptin receptor in neurons phenocopy the overt obesity observed in ob/ob and db/db mice,^22^ we reasoned that the obesogenic action of PI3Kγ may also depend on its activity in neurons. This hypothesis is consistent with a study showing that the intracerebroventricular (ICV) administration of a low dosage of PI3K inhibitors induced adipose tissue lipolysis.^12^ However, such pharmacological PI3K ICV inhibition was not specific for PI3Kγ.^12^ Therefore, the cell type where PI3Kγ activity promotes adiposity and metabolic dysfunction remains unknown.

In this study, we generated mice lacking PI3Kγ conditionally in neurons and investigated their metabolic phenotype in a model of diet-induced obesity, insulin resistance, and MASLD.

## Results

### Generation and characterization of mice with brain-specific ablation of PI3Kγ

**Figure 1:**
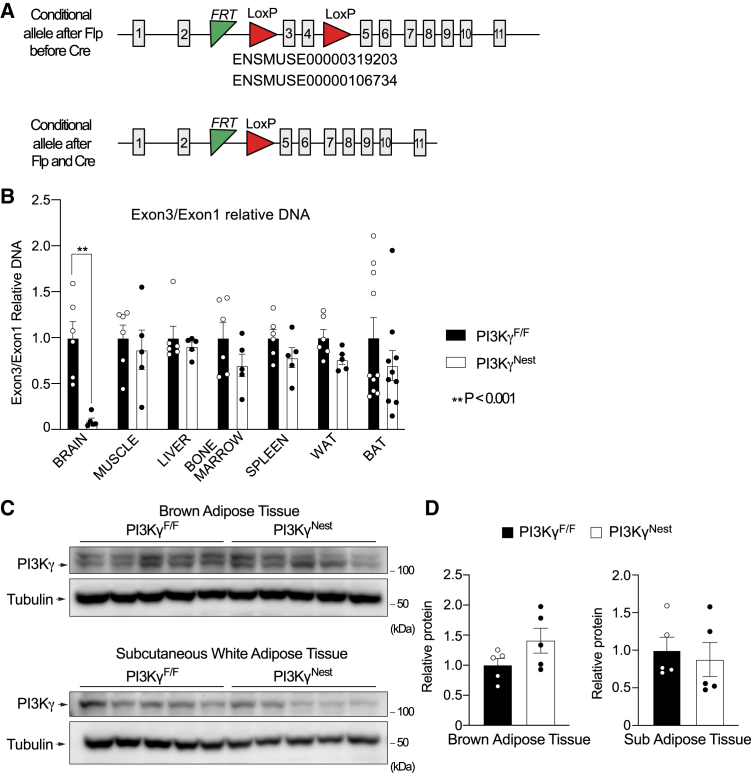
Specific PI3Kγ deletion in PI3Kγ^Nest^ mice (A) Schematic representation of the targeted PIK3CG allele before Cre recombination (PI3Kγ^F/F^ mice) and after Cre recombination (PI3Kγ^Nest^ mice). (B) PIK3CG Exon3/Exon1 relative ratio was measured by real-time qPCR of genomic DNA extracted from the brain, muscle, liver, bone marrow, spleen, white adipose tissue (WAT), and brown adipose tissue (BAT) of PI3Kγ^F/F^ and PI3Kγ^Nest^ mice. n = 5–10. (C) Immunoblots analysis of PI3K abundance in brown adipose tissue and white adipose tissue. (D) Quantification of the immunoblots in C. n = 5 Data are represented as mean ± SEM. Statistical analysis was performed using Mann-Whitney. *p* < 0.01 (∗∗).

To investigate the role of brain PI3Kγ activity in diet-induced obesity, we have generated neuron-specific PI3Kγ knockout mice using Cre-LoxP technology. We crossed PI3Kγ^F/F^ mice, with exons 3 and 4 of the PIK3CG gene flanked by *LoxP* sites,^13^ with mice expressing the Cre recombinase under the control of the Nestin promoter (Nestin-CRE) to generate PI3Kγ^Nest^ mice (Figure 1A). Genomic DNA was extracted from different tissues of PI3Kγ^F/F^ mice PI3Kγ^Nest^ mice, and the efficiency and specificity of Cre recombination were evaluated by performing real-time quantitative PCR analysis of the PIK3CG gene exon-3/exon-1 ratio (Figure 1B). We observed about a 90% reduction of exon-3/exon-1 ratio in the brain of PI3Kγ^Nest^ mice relative to PI3Kγ^F/F^ mice, indicating efficient deletion of PIK3CG exon-3 in neurons of PI3Kγ^Nest^ mice. No other tissue showed a comparable reduction of the exon-3/exon-1 ratio. We observed for some mice a partial reduction of the exon-3/exon-1 ratio in brown adipose tissue, but this reduction was partial and not statistically significant. Immunoblot analysis of white and brown adipose tissues also indicates that PI3Kγ protein abundance is not affected in fat tissues of PI3Kγ^Nest^ mice (Figures 1C and 1D).

These results indicate that PIK3CG exon-3 was nearly completely ablated in neurons of PI3Kγ^Nest^ mice, with no comparable deletion in other tissues.

### PI3Kγ^Nest^ mice show reduced linear growth and are protected from diet-induced obesity

**Figure 2:**
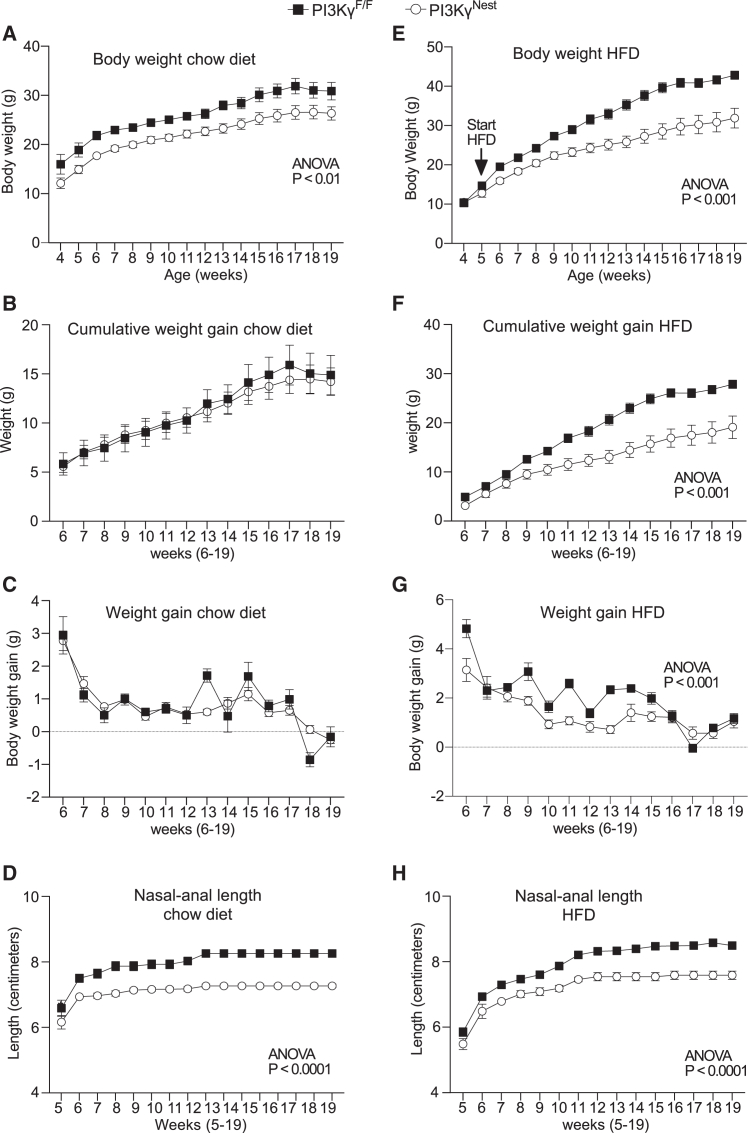
PI3Kγ^Nest^ mice display reduced linear growth and are protected from HFD-induced obesity (A) Body weight growth curves of PI3Kγ^F/F^ and PI3Kγ^Nest^ mice kept on a chow diet. (B) Cumulative weight gain of PI3Kγ^F/F^ and PI3Kγ^Nest^ mice kept on a chow diet. (C) Weekly weight gain of PI3Kγ^F/F^ and PI3Kγ^Nest^ mice kept on a chow diet. (D) Linear growth curves, expressed as naso-anal length, of PI3Kγ^F/F^ and PI3Kγ^Nest^ mice kept on a chow diet. (E) Body weight growth curves for PI3Kγ^F/F^ and PI3Kγ^Nest^ mice kept on HFD. (F) Cumulative weight gain of PI3Kγ^F/F^ and PI3Kγ^Nest^ mice kept on HFD. (G) Weekly weight gain of PI3Kγ^F/F^ and PI3Kγ^Nest^ mice on HFD. (H) Linear growth curves, expressed as naso-anal length, of PI3Kγ^F/F^ and PI3Kγ^Nest^ mice on HFD. *n* = 7 PI3Kγ^F/F^ mice and *n* = 13 PI3Kγ^Nest^ mice for A-D. *n* = 12 PI3Kγ^F/F^ mice and *n* = 7 PI3Kγ^Nest^ mice for E-H. Data are represented as mean ± SEM. Statistical analysis was performed using repeated-measures (RM) two-way ANOVA.

Compared to littermate PI3Kγ^F/F^ mice, PI3Kγ^Nest^ mice kept on a chow diet showed a significantly reduced body weight growth curve associated with reduced body weight at weaning (Figure 2A). However, PI3Kγ^F/F^ mice and PI3Kγ^Nest^ mice showed similar cumulative weight gain and weekly weight gain over a 15-week measurement period (Figures 2B and 2C). Body length growth curves were also significantly reduced in PI3Kγ^Nest^ mice compared to PI3Kγ^F/F^ mice (Figure 2D), indicating that the reduced body weight observed in PI3Kγ^Nest^ mice kept on a chow diet can be mostly explained by reduced linear growth with most differences developing before weaning and in the first weeks after weaning. These results have been qualitatively reproduced in an independent cohort, which showed more modest differences in weight growth and linear growth curves (Figures S1A–S1D).

PI3Kγ^Nest^ mice were largely protected from diet-induced obesity. Indeed, when kept on an obesogenic high-fat diet (HFD), PI3Kγ^Nest^ mice showed a marked reduction in body weight growth curves and weight gain compared to littermate PI3Kγ^F/F^ mice (Figures 2E–2G). However, differences in linear growth between PI3Kγ^Nest^ mice and littermate PI3Kγ^F/F^ control mice kept in HFD were similar to those observed in mice kept on a chow diet (Figures 2D and 2H). The substantial protection of PI3Kγ^Nest^ mice from diet-induced obesity was reproduced in an independent cohort (Figures S1E–S1H).

We conclude that PI3Kγ^Nest^ mice display two distinct phenotypes: a reduced linear growth that was independent of the type of diet and a substantial resistance to diet-induced obesity, which was not associated with the reduced linear growth.

### PI3Kγ^Nest^ mice are protected from HFD-induced insulin resistance and fatty liver

**Figure 3:**
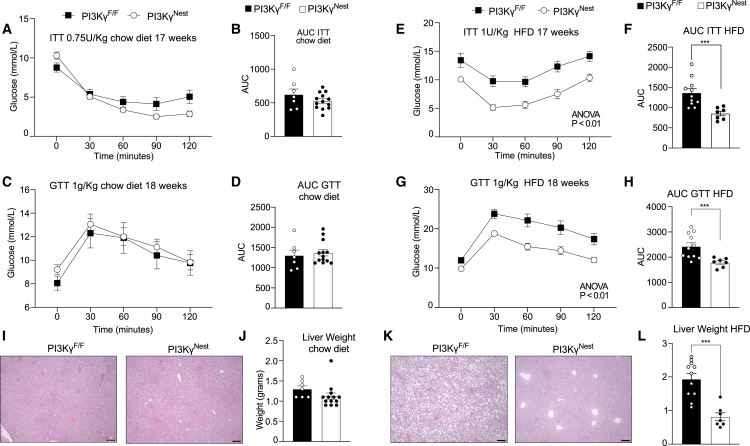
PI3Kγ^Nest^ mice are protected from HFD-induced insulin resistance and fatty liver (A) Insulin tolerance test (ITT) of 17-week-old PI3Kγ^F/F^ and PI3Kγ^Nest^ mice kept on a chow diet. Mice were fasted for 4 h and injected intraperitoneally with 0.75 IU of insulin per Kg of body weight. (B) Area under the curve of the ITT in A. (C) Glucose tolerance test (GTT) of 18-week-old PI3Kγ^F/F^ and PI3Kγ^Nest^ mice on a chow diet. Mice were fasted for 4 h and injected intraperitoneally with 1 g of glucose per kg of body weight. (D) Area under the curve of the GTT in C. (E) Insulin tolerance test (ITT) of 17-week-old PI3Kγ^F/F^ and PI3Kγ^Nest^ mice kept on a high-fat diet (HFD). Mice were fasted for 4 h and injected intraperitoneally with 1 IU of insulin per Kg of body weight. (F) Area under the curve of the ITT in E. (G) Glucose tolerance test (GTT) of 18-week-old PI3Kγ^F/F^ and PI3Kγ^Nest^ mice on HFD. Mice were fasted for 4 h and injected intraperitoneally with 1 g of glucose per kilogram of body weight. (H) Area under the curve of the GTT in (G). (I) Representative images of liver histology of the chow-fed PI3Kγ^F/F^ and PI3Kγ^Nest^ mice. (J) Liver weight for the mice in (I). (K) Representative images of liver histology of HFD-fed PI3Kγ^F/F^ and PI3Kγ^Nest^ mice. (L) Liver weight for the mice in K. n = 7 PI3Kγ^F/F^ mice and *n* = 13 PI3Kγ^Nest^ mice for A-D, I-J and *n* = 11 PI3Kγ^F/F^ mice and *n* = 7 PI3Kγ^Nest^ mice for E–H, K-L. Scale Bar 100 μm for I, K. Data are represented as mean ± SEM. Statistical analysis was performed using repeated-measures (RM) two-way ANOVA for A, C, E, G, and Mann-Whitney for B, D, F, H, J, L. P < 0.001 (∗∗∗).

On a chow diet, PI3Kγ^Nest^ mice and PI3Kγ^F/F^ mice displayed similar insulin tolerance, as differences were marginal and not statistically significant by repeated measures ANOVA (Figures 3A and 3B). Glucose tolerance was virtually identical in PI3Kγ^Nest^ mice (Figures 3C and 3D). By contrast, when kept on HFD, PI3Kγ^Nest^ mice showed a substantial improvement in insulin tolerance (Figures 3E and 3F) and glucose tolerance, compared to PI3Kγ^F/F^ control mice (Figures 3G and 3H). The marked protection of PI3Kγ^Nest^ mice from diet-induced insulin intolerance and glucose intolerance was reproduced in an independent cohort (Figure S2).

Liver histology and measurements of liver weight showed that whereas PI3Kγ^F/F^ mice developed overt hepatosteatosis when placed on HFD, PI3Kγ^Nest^ mice were virtually completely protected from the effects of HFD on hepatic lipid accumulation (Figures 3I–3L). The nearly complete protection of PI3Kγ^Nest^ mice from diet-induced hepatosteatosis was replicated in an independent cohort of mice (Figure S3).

### Resistance to diet-induced obesity of PI3Kγ^Nest^ mice depends on conditional PI3Kγ deletion

The reduced linear growth observed in PI3Kγ^Nest^ mice compared to littermate PI3Kγ^F/F^ control mice was unexpected. Indeed, whole-body PI3Kγ knockout mice (PI3Kγ^−/−^) do not show reduced linear growth compared to WT controls.^8^ Previous studies reported reduced linear growth in Nestin-CRE mice^23^ indicating that the reduced linear growth observed in PI3Kγ^Nest^ mice could be an artifact of the Cre expression in neurons. To discriminate between the metabolic effect of the Cre expression in neurons and the effect of PI3Kγ ablation in neurons, we crossed PI3Kγ^F/+^-Cre^-^ mice with PI3Kγ^F/+^-Cre^+^ mice to obtain four groups of littermate mice: WT; Nestin-CRE (not *loxP* floxed); PI3Kγ^F/F^; and PI3Kγ^Nest^ mice. Due to the low productivity of this breeding, we could not generate a sufficient number of littermate mice to perform the experiments with all littermate controls. Therefore, we used these mice as breeders to generate two parallel age-matched groups of littermate experimental mice (Figure S4). Littermate-group 1 consisted of littermates PI3Kγ^F/F^ and PI3Kγ^Nest^ mice, and littermate-group 2 consisted of WT and Nestin-CRE littermate mice. The four genotypes were age-matched and generated from littermate breeders. All mice were placed on HFD, and experiments were performed in parallel at the same time for all mice.

**Figure 4:**
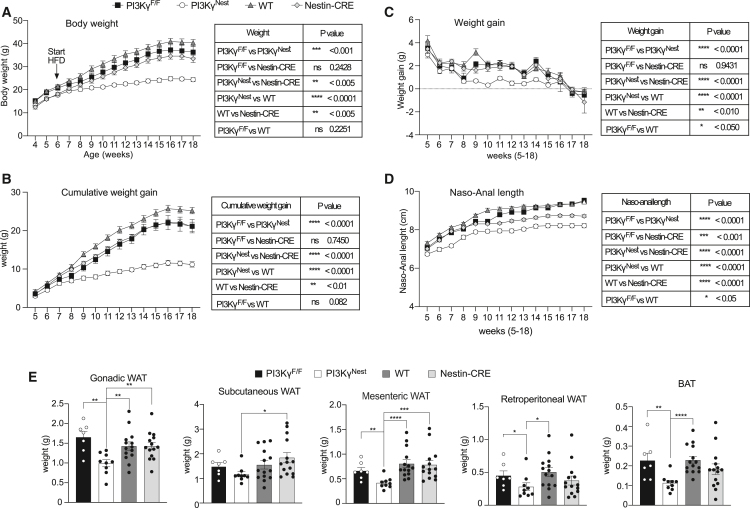
Loss of PI3Kγ in neurons protects mice from HFD-induced obesity (A) Growth curves for PI3Kγ^F/F^, PI3Kγ^Nest^, WT, and Nestin-CRE mice placed on an obesogenic high-fat diet (HFD). (B) Cumulative weight gain of PI3Kγ^F/F^, PI3Kγ^Nest^, WT, and Nestin-CRE mice on HFD. (C) Weekly weight gain of PI3Kγ^F/F^, PI3Kγ^Nest^, WT, and Nestin-CRE mice on HFD. (D) Linear growth curves, expressed as naso-anal length, of PI3Kγ^F/F^, PI3Kγ^Nest^, WT, and Nestin-CRE mice on HFD. (E) Adipose tissue fat pad weights for the mice in A. *n* = 7 PI3Kγ^F/F^ mice, *n* = 9 PI3Kγ^Nest^ mice, *n* = 14 mice for WT and Nestin-CRE for all panels. Data are represented as mean ± SEM. Statistical analysis was performed using repeated-measures (RM) two-way ANOVA for A-D and Mann-Whitney for E. *p* < 0.05 (∗∗), *p* < 0.01 (∗∗), *p* < 0.001 (∗∗∗), *p* < 0.0001 (∗∗∗∗).

Nestin-CRE mice showed significant protection from diet-induced obesity compared to littermate WT mice (Figure 4A). However, the weight gain of Nestin-Cre mice was similar to the one of PI3Kγ^F/F^ mice (Figures 4B and 4C), and PI3Kγ^Nest^ mice showed marked protection from diet-induced obesity compared to all the control groups, including Nestin-CRE mice (Figures 4A and 4B). As expected, Nestin-CRE showed reduced linear growth compared to WT littermates and also compared to PI3Kγ^F/F^ mice (Figure 4D), indicating that the reduced linear growth of PI3Kγ^Nest^ mice is an artifact of the Cre expression in neurons.^23^ PI3Kγ^Nest^ mice were significantly shorter than Nestin-CRE mice, indicating a possible interaction between the effects of Cre expression in neurons on linear growth and the metabolic effects specific for PI3Kγ ablation in neurons. Measurement of fat pad weights shows that PI3Kγ^Nest^ mice display reduced adiposity compared to all groups, including Nestin-CRE mice (Figure 4E).

Overall, we conclude that the protection from diet-induced obesity observed in PI3Kγ^Nest^ mice is mostly due to the specific PI3Kγ ablation in neurons. The reduced linear growth observed in PI3Kγ^Nest^ mice is an artifact of the Cre expression in neurons with possible interaction with the metabolic phenotype caused by the loss of PI3Kγ activity in neurons. This interpretation is consistent with the fact that PI3Kγ^−/−^ mice display marked protection from diet-induced obesity without showing a defect in linear growth.^8^^,^^9^

### PI3Kγ ablation in neurons protects mice from HFD-induced insulin resistance and glucose intolerance

**Figure 5:**
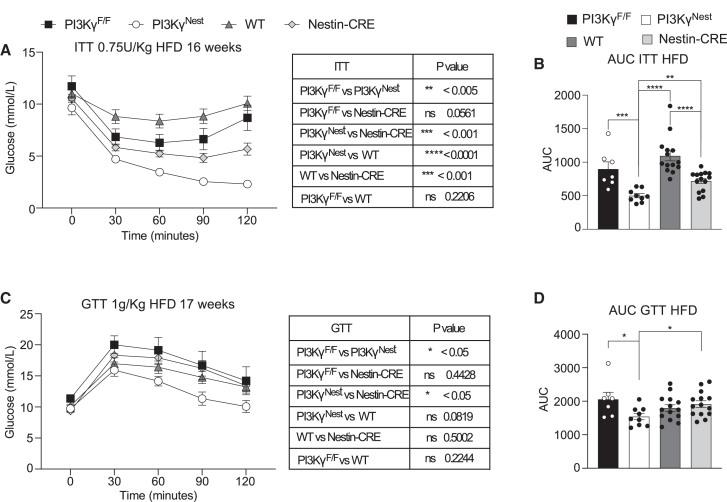
PI3Kγ ablation in neurons protects mice from HFD-induced insulin resistance (A) Insulin tolerance test (ITT) of 16-week-old PI3Kγ^F/F^, PI3Kγ^Nest^, WT, and Nestin-CRE mice kept on a high-fat diet (HFD). Mice were fasted for 4 h and injected intraperitoneally (i.p.) with 0.75 IU of insulin per Kg of body weight. (B) Area under the curve of the ITT in A. (C) Glucose tolerance test (GTT) of 17-weeks-old PI3Kγ^F/F^, PI3Kγ^Nest^, WT, and Nestin-CRE mice kept on HFD. Mice were fasted for 4 h and injected intraperitoneally (i.p.) with 1 g of glucose per kg of body weight. (D) Area under the curve of the GTT in C. n = 7 PI3Kγ^F/F^ mice, *n* = 9 PI3Kγ^Nest^ mice, *n* = 14 mice for WT and Nestin-CRE. Data are represented as mean ± SEM. Statistical analysis was performed using repeated-measures (RM) two-way ANOVA for A and C and Mann-Whitney for B and D. *p* < 0.05 (∗), *p* < 0.01 (∗∗), *p* < 0.001 (∗∗∗), *p* < 0.0001 (∗∗∗∗).

Nestin-CRE mice showed significantly improved insulin tolerance compared to WT mice but similar insulin tolerance to PI3Kγ^F/F^ mice (Figures 5A and 5B). However, PI3Kγ^Nest^ mice showed a significantly improved insulin tolerance compared to all groups, including Nestin-CRE mice (Figures 5A and 5B).

In the glucose tolerance test, Nestin-CRE mice showed similar glucose excursions to PI3Kγ^F/F^ mice and WT mice (Figures 5C and 5D). By contrast, PI3Kγ^Nest^ mice showed the lowest glucose excursion, which was statistically significant compared to Nestin-CRE mice and PI3Kγ^F/F^ mice (Figures 5C and 5D). We conclude that although Nestin-CRE mice display a significant metabolic phenotype, ablation of PI3Kγ in neurons protects mice from HFD-induced insulin resistance.

The combined average of data from all cohorts confirms the specific protection of PI3Kγ^Nest^ mice from diet-induced obesity and glucose intolerance (Figures S5 and S6).

### PI3Kγ ablation in neurons protects mice from HFD-induced metabolic inflammation and steatosis

**Figure 6:**
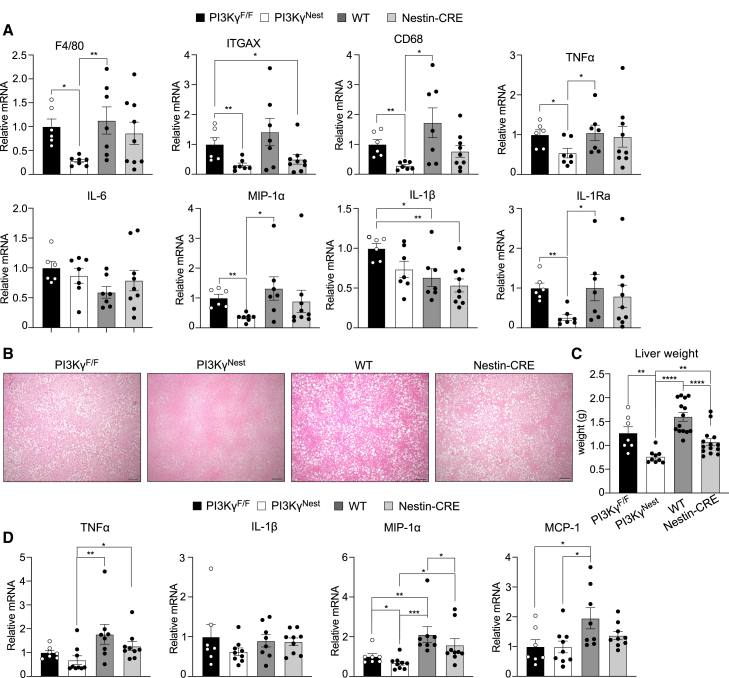
Mice lacking PI3Kγ in neurons are protected from HFD-driven fatty liver and display reduced metabolic inflammation (A) Expression of inflammation markers in epididymal adipose tissues from PI3Kγ^F/F^, PI3Kγ^Nest^, WT, and Nestin-CRE mice kept on an obesogenic high-fat diet (HFD). (B) Representative images of liver histology of PI3Kγ^F/F^, PI3Kγ^Nest^, WT, and Nestin-CRE mice kept on HFD. (C) Liver weight for the mice in B. (D) Expression of inflammation markers in the livers of the mice from above. n = 6–9 for A, B, and D; n = 7–14 for B and C. Scale Bar 100 μm. Data are represented as mean ± SEM. Statistical analysis was performed using Mann-Whitney analysis. *p* < 0.05 (∗), *p* < 0.01 (∗∗), *p* < 0.001 (∗∗∗) *p* < 0.0001 (∗∗∗∗).

The improved insulin sensitivity observed in mice with whole-body ablation of PI3Kγ was associated with reduced metabolic inflammation and hepatosteatosis^8^^,^^9^; a phenotype that was associated with their protection from diet-induced obesity.^8^^,^^13^ Real-time qPCR mRNA profiling of proinflammatory markers in epididymal adipose tissue of WT, Nestin-CRE, PI3Kγ^F/F^, and PI3Kγ^Nest^ mice showed a general reduction of pro-inflammatory gene expression in PI3Kγ^Nest^ mice (Figure 6A). The mRNA abundances of the macrophage markers F4/80, ITGAX, and CD68 and the M1-macrophage cytokines TNFα, MIP1α, and IL1Ra were significantly downregulated in PI3Kγ^Nest^ mice compared to PI3Kγ^F/F^ and WT mice (Figure 6A). The average mRNA abundance of PI3Kγ^Nest^ mice inflammatory markers was also reduced compared to Nestin-CRE mice, but this difference did not achieve statistical significance. However, the expression of F4/80, TNFα, IL-6, IL-1β, and IL-1Ra in Nestin-CRE mice was similar to that of their littermate WT control mice. Compared to WT mice, Nestin-CRE mice showed a reduced abundance of ITGAX and CD68, but this difference was not statistically significant.

Liver histology showed that PI3Kγ^Nest^ mice were largely protected from diet-induced steatosis compared to all groups, including Nestin-CRE mice (Figure 6B). Nestin-CRE mice showed a trend toward less severe steatosis compared to littermate WT mice, but this difference was not comparable to the almost complete protection observed in PI3Kγ^Nest^ mice (Figure 6B). Consistently with the histology data PI3Kγ^Nest^ mice showed a significantly reduced liver weight compared to all the groups, including Nestin-CRE mice (Figure 6C). Real-time qPCR mRNA profiling of proinflammatory markers from the livers above showed a significantly reduced abundance of TNFα and MIP-1α mRNA in the livers of PI3Kγ^Nest^ mice (Figure 6D). Compared to WT littermates, Nestin-CRE mice showed a small but statistically significant reduction in MIP-1α mRNA in their livers. However, MIP-1α mRNA in the livers of Nestin-CRE mice was significantly higher than the one measured in PI3Kγ^Nest^ mice (Figure 6D).

Overall, our data indicate that Nestin-CRE mice display a partial improvement in hepatosteatosis without an obvious improvement in metabolic inflammation. However, PI3Kγ^Nest^ mice were almost completely protected from HFD-induced hepatic steatosis and showed a significant reduction in the expression of inflammatory markers in their adipose tissue and livers. We conclude that ablation of PI3Kγ in neurons largely prevents the development of fatty liver and significantly reduces metabolic inflammation in a mouse model of diet-induced obesity.

### PI3Kγ ablation in neurons increases adipose tissue HSL phosphorylation at PKA sites

**Figure 7:**
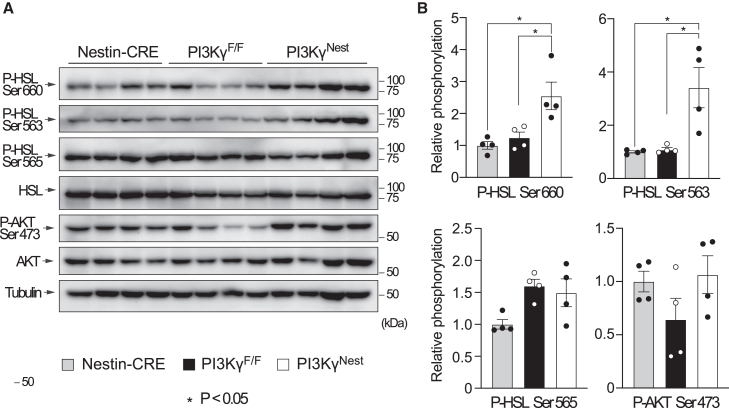
Loss of PI3Kγ increases adipose tissue HSL phosphorylation at PKA sites (A) Immunoblot analysis of HSL and AKT phosphorylation of subcutaneous adipose tissue samples from Nestin-CRE, PI3Kγ^F/F^, and PI3Kγ^Nest^ mice described in Figure 4. (B) Quantification of the immunoblots in A. n = 4 for Nestin-CRE, PI3Kγ^F/F^, and PI3Kγ^Nest^ mice. Data are represented as mean ± SEM. Statistical analysis was performed using Mann-Whitney analysis. *p* < 0.05 (∗).

Mice with whole-body deletion of PI3Kγ show increased phosphorylation of hormone-sensitive lipase (HSL) at protein kinase A (PKA) sites,^8^^,^^13^ and a similar increase in HSL was observed after intracerebroventricular administration of PI3K inhibitors.^12^ Therefore, we compared HSL phosphorylation in white adipose tissue from PI3Kγ^Nest^ mice, PI3Kγ^F/F^ mice, and Nestin-CRE mice (Figure 7). The results show that PI3Kγ^Nest^ mice display significantly increased adipose tissue HSL phosphorylation, specifically at the PKA sites serine-563 and serine-660, compared to PI3Kγ^F/F^ mice and Nestin-CRE mice. PI3Kα and PI3Kβ expression was not altered in adipose tissues, livers, or muscles of PI3Kγ^Nest^ mice (Figure S7), and AKT phosphorylation in adipose tissue was similar between all the groups (Figure 7).

We conclude that ablation of PI3Kγ in neurons results in increased adipose tissue HSL phosphorylation at PKA sites.

## Discussion

Mice with whole-body deletion of PI3Kγ are protected from diet-induced obesity, a phenotype associated with increased energy expenditure and PKA-dependent HSL phosphorylation in adipose tissue.^8^^,^^9^^,^^12^^,^^13^^,^^14^ The results presented in this study indicate that PI3Kγ action in diet-induced obesity depends on its activity in neurons.

Indeed, we have found that PI3Kγ^Nest^ mice, lacking PI3Kγ in neurons, are protected from diet-induced obesity, hepatosteatosis, and insulin resistance to a similar extent to the one reported in previous studies for mice with whole-body deletion of PI3Kγ.^8^^,^^9^^,^^13^^,^^14^

Systemic administration of pan-PI3K inhibitors reduced adiposity in rodents and primates, an effect that was associated with the inhibition of insulin signaling in the adipocyte.^15^^,^^24^ However, systemic pan-PI3K inhibition is also associated with toxic side effects^25^ and hyperinsulinemia, which reduces the efficacy of PI3K-targeted therapies.^7^

PI3Kγ is dispensable for insulin signaling in the adipocytes and hepatocytes, which instead depends on the redundant activities of PI3Kα and PI3Kβ, whose inhibition drives the feedback responsible for increased insulin secretion.^18^^,^^20^ Because PI3Kγ^Nest^ mice do not develop insulin resistance but display improved insulin tolerance and glucose tolerance, pharmacological inhibition of PI3Kγ in neurons may be an effective anti-obesogenic strategy.

The specific type of neurons responsible for the action of PI3Kγ in adiposity remains to be identified. A previous study using intracerebroventricular administration of PI3K inhibitors and a cell line proposed that redundant PI3Kγ and PI3Kβ activities within the hypothalamus inhibit α-MSH-dependent sympathetic drive.^12^ Thus, the anti-obesogenic action of PI3Kγ may depend on its activity in neurons expressing the αMSH receptor MC4R controlling HSL activity in adipocytes, possibly through increased sympathetic activity in adipose tissue. However, any neuron expressing a G-protein coupled receptor implicated in controlling adiposity and PI3Kγ could be a good candidate. PI3Kγ is expressed in different types of neurons and has been implicated in a variety of neuronal functions, including odorant signaling,^26^ antinociceptive response,^27^ cannabidiol anticonvulsant and neuroprotective effects,^28^ GLP-2 signaling^29^; itching signal transmission,^30^ ADHD,^31^ μ-opioid receptor signaling,^32^ morphine analgesia^33^ anti-hyperalgesia,^34^ and NMDA-signaling.^35^ Furthermore, the receptors for glucagon-like peptide-1 (GLP-1), glucose-dependent insulinotropic polypeptide (GIP), and glucagon (GCGR), which are the targets of modern antio-besogenic drugs, are all G-protein coupled receptors, whose signal may be regulated by PI3Kγ, as suggested by cell culture studies.^36^^,^^37^ Therefore, in the future, it will be important to investigate the action of PI3Kγ in GLP-1R, GIPR, and GCGR-positive neurons and whether PI3Kγ inhibition in these cells potentiates cAMP signaling.

In conclusion, our study indicates that the metabolic action of PI3Kγ depends on its activity in neurons. This metabolic effect protected mice from HFD-mediated promotion of hepatocellular carcinoma,^19^ suggesting that central PI3Kγ inhibition may contribute to a more favorable metabolic and therapeutic outcome for cancer patients who are overweight. Although the relevance of our findings to humans remains to be demonstrated, possible effects on adipose tissue metabolism of PI3Kγ inhibitors capable of crossing the brain-blood barrier should be considered.

### Limitations of the study

The major limitation of this study is that Cre expression in neurons has a metabolic phenotype, which complicates data interpretation.^23^ However, PI3Kγ^Nest^ mice showed reduced adiposity, steatosis, and improved insulin sensitivity also compared to Nestin-CRE mice, indicating that the metabolic phenotype of PI3Kγ^Nest^ mice depends on PI3Kγ deletion. PI3Kγ^Nest^ mice showed a partial deletion in BAT of some mice, and therefore, we cannot exclude a contribution of PI3Kγ activity in brown adipocytes to their leaner phenotype. However, while virtually all PI3Kγ^Nest^ mice were protected from diet-induced obesity, PIK3CG’s Exon-3/Exon-1 ratio, was not consistently reduced in the BAT of all mice. Therefore, the metabolic phenotype of PI3Kγ^Nest^ mice must depend on the loss of PI3Kγ in neurons.

Finally, our study cannot conclude to which extent the leaner phenotype of PI3Kγ^Nest^ mice depends on differences in food intake or energy expenditure. However, we previously reported that the leaner phenotype of whole-body PI3Kγ knockout mice was not associated with a measurable reduction in food intake.^8^ Altogether, our results indicate that the metabolic action of PI3Kγ in diet-induced obesity depends on its activity in neurons.

## Resource availability

### Lead contact

Further information and requests for resources and reagents should be directed to and will be fulfilled by the Lead Contact, Giovanni Solinas (giovanni.solinas@wlab.gu.se).

### Materials availability

This study did not generate new unique reagents.

### Data and code availability

• Data reported in this paper will be shared by the lead contact upon request.
• This paper does not report original code.
• Any additional information required to reanalyze the data reported in this paper is available from the lead contact upon request.

## Acknowledgments

This study is supported by funding from the 10.13039/501100004359Swedish Research Council 2022-01033, the 10.13039/501100002794Cancerfonden 23 2829 Pj and by 10.13039/501100008550Diabetesfonden DIA2022-753 to G.S.

## Author contributions

G.S. designed research. A.A., A.M., A.U.G., A.C.G., V.R.R.S., J.R., and B.B. performed experiments and data analysis. D.R. and M.L. provided unique tools. A.A., A.M., A.U.G., A.C.G., V.R.R.S., J.R., B.B., and G.S. interpreted the results. G.S. wrote the manuscript. All the authors read and approved the manuscript.

## Declaration of interests

The authors declare no conflict of interest for this manuscript.

## STAR★Methods

### Key resources table

**Table:** 

REAGENT or RESOURCE	SOURCE	IDENTIFIER
**Antibodies**		

AKT Phospho (Thr308)	Cell Signaling	4056; RRID:AB_331163
AKT Phospho (Ser373) XP	Cell Signaling	4060; RRID:AB_2315049
AKT	Cell Signaling	9272; RRID:AB_329827
HSL Phospho (Ser563)	Cell Signaling	4139; RRID:AB_2135495
HSL Phospho (Ser660)	Cell Signaling	4126; RRID:AB_490997
HSL Phospho (Ser565)	Cell Signaling	4137; RRID:AB_2135498
HSL	Cell Signaling	4107; RRID:AB_2296900
Tubulin ?/?	Cell Signaling	2148; RRID:AB_2288042

**Chemicals, peptides, and recombinant proteins**		

Insulin	Humalog Lilly	VL7510
Glucose	Acros	410955000
BSA	Fisher Scientific	BP9702
Proteinase K	New England Biolabs	P8107S

**Critical commercial assays**		

ECL-anti rabbit IgG HRP	GE Healthcare	NA934V
Immobilon Western Chemiluminescence HRP substrate	Millipore	WBKLS0500
Hematoxylin Solution (Mayer's, Modified)	Abcam	AB220365
Eosin	Sigma	HT110116
Glucose strips	Contour Next	Ascensia
ImProm-II™ Reverse Transcriptase	Promega	A3803
SsoAdvanced Universal SYBR® Green Supermix	Bio-Rad	1725270

**Deposited data**		

Immunoblots images	This paper	https://doi.org/10.17632/pmbr5t3wxs.1

**Experimental models: Organisms/strains**		

Nestin-CRE	C57BL/6 background	RRID: IMSR_JAX:003771
*PI3K*γ^*F/F*^	C57BL/6 background	From EUCOMM
*PI3K*γ^*Nest*^	C57BL/6 background	From this study

**Oligonucleotides**		

Exon 1 pik3cg	GCCCCGGGTAGG TCTAGA	GTGATGCGGAGGAGGATCATT
Exon 3 pik3cg	TTTGAACCGTACCACGACAGT	CCACGCTTCAGCAGGAATCT
Cyclophilin; GenBank: NM_008907	ATG GTC AAC CCC ACC GTG T (For)	TTT CTG CTG TCT TTG GAA CTT TGT C (Rev)
F4/80; GenBank: NM_010130.4	CTT TGG CTA TGG GCT TCC AGT C (For)	GCA AGG AGG ACA GAG TTT ATC GTG (Rev)
Itgax (CD11c); GenBank: NM_021334.2	CTG GAT AGC CTT TCT TCT GCT G (For)	GCA CAC TGT GTC CGA ACT C (Rev)
CD68; GenBank: DQ167574.1	CCT CGC CTA GTC CAA GGT C (For)	GGA TTC GGA TTT GAA TTT GGG CT (Rev)
TNF-α; GenBank: NM_013693.2	CCC CAA AGG GAT GAG AAG TT (For)	CTC CTC CAC TTG GTG GTT TG (Rev)
IL-6; GenBank: NM_031168	TCC TAC CCC AAT TTC CAA TGC TC (For)	TTG GAT GGT CTT GGT CCT TAG CC (Rev)
MIP-1α; GenBank: NM_011337.2	TTC TCT GTA CCA TGA CAC TCT GC (For)	CGT GGA ATC TTC CGG CTG TAG (Rev)
IL-1β; GenBank: NM_008361.3	GCA ACT GTT CCT GAA CTC AAC T (For)	TCT TTT GGG GTC CGT CAA CT (Rev)
IL-1Ra; GenBank: NM_001039701	AAA TCT GCT GGG GAC CCT AC (For)	TCT TCT AGT TTG ATA TTT GGT CCT TG (Rev)

**Software and algorithms**		

Image Lab software (version 5.2.1)	Bio-Rad	http://www.bio-rad.com/en-us/product/image-lab-software
Graph Pad Prism 7.0	Graph Pad Software	N/A
AxioVision Software	Imaging Sofware	Carl Zeiss Microscopy

### Experimental model and study participant details

#### Mice

All mice studies were approved by the Ethics Committee on Animal Care Use in Gothenburg, Sweden. Study approval numbers: Dnr 5.8.18-14666/2018 and Dnr 5.8.18-06398/2021. Only male mice were used in this study. Mice were maintained at the specific-pathogen-free EBM facility of the University of Gothenburg under a 12-hour light /12-hour dark cycle at a room temperature of about 22°C. PI3Kγ^F/F^ mice were obtained by crossing Pik3cg<tm1a(EUCOMM)Wtsi>/Wtsi mice [from the European Conditional Mouse Mutagenesis Program (EUCOMM)] with mice expressing the Flippase (FLP) recombinase under the constitutive *actin* promoter{Breasson, 2017, 28720716}. PI3Kγ^Nest^ were generated by crossing PI3Kγ^F/F^ mice with transgenic mice (from the Jackson Laboratory, B6.Cg-Tg(Nes-cre)1Kln/J) expressing the Cre recombinase under the control of the rat nestin promoter and enhancer. Nestin CRE were obtained from Jackson labs. All mice are in pure C57BL/6J genetic background.

### Method details

#### Tissue-specific deletion

To evaluate the tissue-specific PI3Kγ deletion, genomic DNA was extracted from different tissues of PI3Kγ^F/F^ and PI3Kγ^Nest^ mice. Tissue pads were digested in 90 μl of digestion buffer (100mM TRIS-HCl, 200mM NaCl, 5 mM EDTA, 0,2% SDS) with proteinase K (2 μl/ml, New England Biolabs p8107) for 2-3 hours while shaking. 90 μl of distilled water were added and the samples were centrifuged for 5 minutes at 10 000 rpm to remove tissue debris. 120 μl were transferred to a new tube and 240 μl of cold 100% ethanol (kept at -20°C) were added. After mixing by inversion, the precipitated DNA was transferred to a new tube containing 500 μl of 70% ethanol and spun at 10’000 rpm for 5 minutes. The DNA was air-dried (or kept 5 minutes at 37°C) before adding distilled water.

The efficiency and specificity of Cre recombination were assessed by real-time quantitative PCR analysis of the PIK3CG exon-3/exon-1 ratio in the genomic DNA of brain, muscle, liver, bone marrow, spleen, white adipose tissues (WAT), and brown adipose tissue (BAT).

#### In Vivo studies

Mice were weaned between 3 and 4 weeks of age and placed either on chow diet or, for the diet-induced obesity model, on a high-fat diet, 60% of calories from fat (Bio-Serv, F3282), at 6 weeks of age.

For the insulin-tolerance test (ITT), mice were fasted for 4 hours and injected intraperitoneally with 0.75 I.U. (chow diet) or 1 I.U. (high-fat diet) of insulin per Kg of body weight. For the glucose tolerance test (GTT), mice were fasted for either 4 hours and injected intraperitoneally with 1 g of glucose per Kg of body weight. Blood glucose concentrations were measured using a glucometer (Contour Next, Ascensia).

#### Histology

Samples of liver pads were fixed in 10% formalin, embedded in paraffin, cut into 5 μm sections, and stained with hematoxylin (Abcam) and eosin (Sigma). The hematoxylin and eosin-stained sections were imaged at 20X magnification with an AxioImager M1 microscope (Zeiss, Germany).

#### Immunoblot analysis

Protein extracts were resolved by SDS-PAGE electrophoresis (8-10% acrylamide bis-acrylamide 29:1) and then transferred to a PVDF membrane. The membrane was blocked at room temperature in phosphate saline buffer (PBS) with 3% bovine serum albumin (BSA) and 0.3% tween for 1 hour. The primary antibody was incubated at 4°C overnight in PBS with 3% BSA and 0.3% tween; the membranes were then washed three times with a PBS 0.3% tween solution and incubated with a horseradish peroxidase-conjugated secondary antibody (anti-rabbit IgG HRP, GE Healthcare) in PBS 3% BSA and 0.3% tween for 1 hour at room temperature. After three washes in a PBS 0.3% tween solution and incubation with a detection reagent (Immobilon Western Chemiluminescence HRP substrate, Millipore), the signal was acquired using a Biorad ChemiDoc Touch imaging instrument.

#### Real-time PCR RNA profiling

Total RNA was isolated from tissues using the guanidinium thiocyanate-phenol-chloroform extraction method. Complementary DNA was prepared using a reverse transcription kit (Promega), and qPCR was performed using a commercial SYBR green mix (SsoAdvanced Universal SYBR Green Supermix. Bio-Rad) using specific primers and cyclophilin as internal control.

### Quantification and statistical analysis

Data are expressed as means, and error bars indicate standard errors.

Only biological replicates, defined as data from different mice or tissues of different mice, are used for statistical analysis. The exact number of mice for a specific experiment is indicated in the figure legends.

In analyses where two different categorical variables are considered (ITT, GTT, mice weight, cumulative weight gain, weight gain, and naso-anal length), repeated measures (RM) two-way ANOVA was used. Mann-Whitney analysis is used to compare two groups with a single datapoint. All statistical analysis was performed using GraphPad Prism.
