# Supplementary material for: Ablation of PI3Kγ in neurons protects mice from diet-induced obesity MASLD and insulin resistance
Source: iScience. 2024 Dec 9;28(1):111562. doi: 10.1016/j.isci.2024.111562 (PMC11732162; doi:10.1016/j.isci.2024.111562)
Supplement: Document S1. Figures S1–S7 [file mmc1.pdf]

iScience, Volume *28*

Supplemental information

Ablation of PI3Kγ in neurons protects mice from diet-induced obesity MASLD and insulin resistance

Angela Molinaro, Arianna Mazzoli, Andrea Usseglio Gaudi, Amit Chand Gupta, Vagner Ramon Rodrigues Silva, Damien Ramel, Muriel Laffargue, Johan Ruud, Barbara Becattini, and Giovanni Solinas

Figure S1

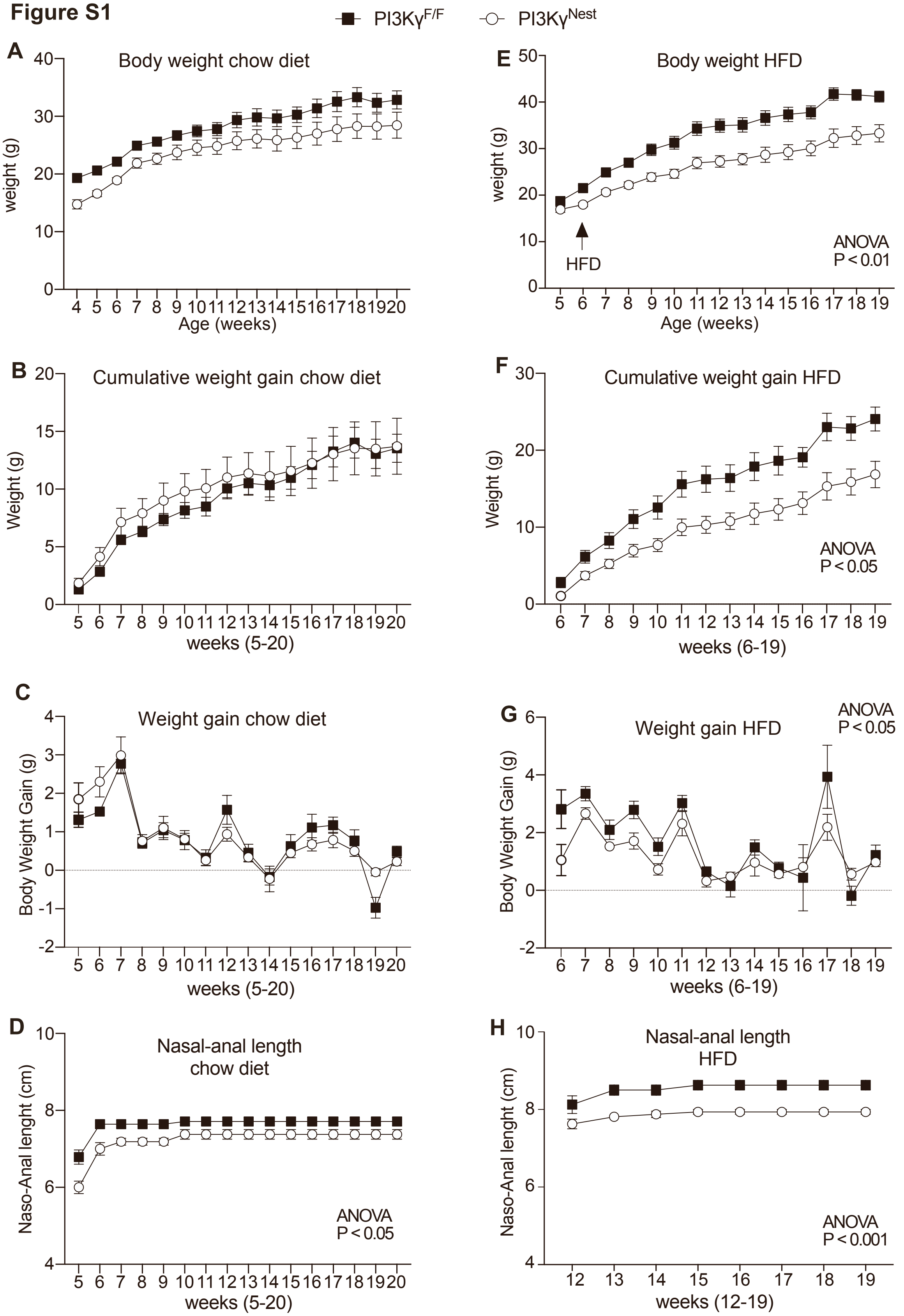
PI3Kγ^F/F
PI3Kγ^Nest
A
Body weight chow diet
weight (g)
Age (weeks)
B
Cumulative weight gain chow diet
Weight (g)
weeks (5-20)
C
Weight gain chow diet
Body Weight Gain (g)
weeks (5-20)
D
Nasal-anal length
chow diet
Naso-Anal lenght (cm)
ANOVA
P < 0.05
weeks (5-20)
E
Body weight HFD
weight (g)
HFD
ANOVA
P < 0.01
Age (weeks)
F
Cumulative weight gain HFD
Weight (g)
ANOVA
P < 0.05
weeks (6-19)
G
Weight gain HFD
ANOVA
P < 0.05
Body Weight Gain (g)
weeks (6-19)
H
Nasal-anal length
HFD
Naso-Anal lenght (cm)
ANOVA
P < 0.001
weeks (12-19)

Figure S1. PI3Kγ^{Nest} mice display reduced linear growth and are protected from diet-induced obesity. Related to Figure 2.

(A) Body weight growth curves of PI3Kγ$^{F/F}$ and PI3Kγ^{Nest} mice kept on a chow diet.

(B) Cumulative weight gain of PI3Kγ$^{F/F}$ and PI3Kγ^{Nest} mice kept on a chow diet.

(C) Weekly weight gain of PI3Kγ$^{F/F}$ and PI3Kγ^{Nest} mice kept on a chow diet.

(D) Linear growth curves, expressed as naso-anal length, of PI3Kγ$^{F/F}$ and PI3Kγ^{Nest} mice kept on a chow diet.

(E) Body weight growth curves for PI3Kγ$^{F/F}$ and PI3Kγ^{Nest} mice kept on HFD.

(F) Cumulative weight gain of PI3Kγ$^{F/F}$ and PI3Kγ^{Nest} mice kept on HFD.

(G) Weekly weight gain of PI3Kγ$^{F/F}$ and PI3Kγ^{Nest} mice on HFD.

(H) Linear growth curves, expressed as naso-anal length, of PI3Kγ$^{F/F}$ and PI3Kγ^{Nest} mice on HFD.

n = 7-8 mice for A-D. n = 8 mice for E-H.

Figure S2

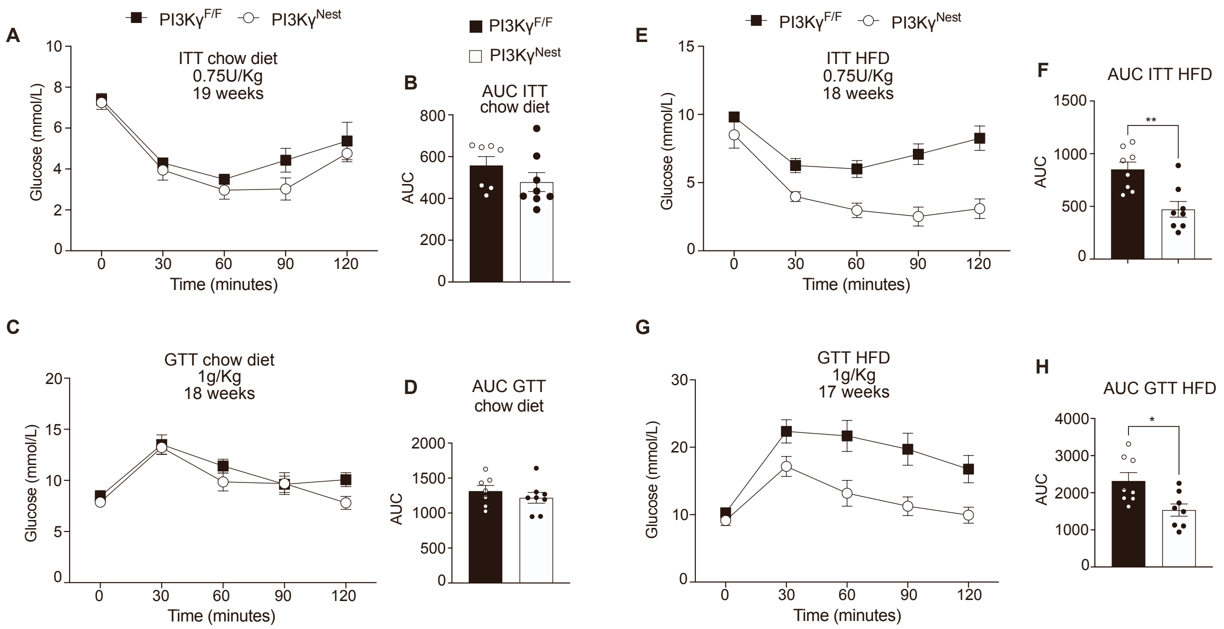

Figure S2. PI3Kγ^{Nest} mice are protected from HFD-induced insulin resistance. Related to Figure 3.

(A) Insulin tolerance test (ITT) of 19-week-old PI3Kγ$^{F/F}$ and PI3Kγ^{Nest} mice kept on a chow diet. Mice were fasted for four hours and injected intraperitoneally with 0.75 IU of insulin per Kg of body weight.

(B) Area under the curve of the ITT in A.

(C) Glucose tolerance test (GTT) of 18-week-old PI3Kγ$^{F/F}$ and PI3Kγ^{Nest} mice on a chow diet. Mice were fasted for 4 hours and injected intraperitoneally with 1 g of glucose per kg of body weight.

(D) Area under the curve of the GTT in C.

(E) Insulin tolerance test (ITT) of 18-week-old PI3Kγ$^{F/F}$ and PI3Kγ^{Nest} mice kept on a high-fat diet (HFD). Mice were fasted for 4 hours and injected intraperitoneally with 1 IU of insulin per Kg of body weight.

(F) Area under the curve of the ITT in E.

(G) Glucose tolerance test (GTT) of 17-week-old PI3Kγ$^{F/F}$ and PI3Kγ^{Nest} mice on HFD. Mice were fasted for 4 hours and injected intraperitoneally with 1 g of glucose per Kg of body weight.

(H) Area under the curve of the GTT in G.

n = 7-8 mice for A-D, n = 8 mice for E-H.

Data are represented as mean ± SEM. Statistical analysis was performed using repeated-measures (RM) two-way ANOVA for A, C, E, G and Mann-Whitney for B, D, F, H.

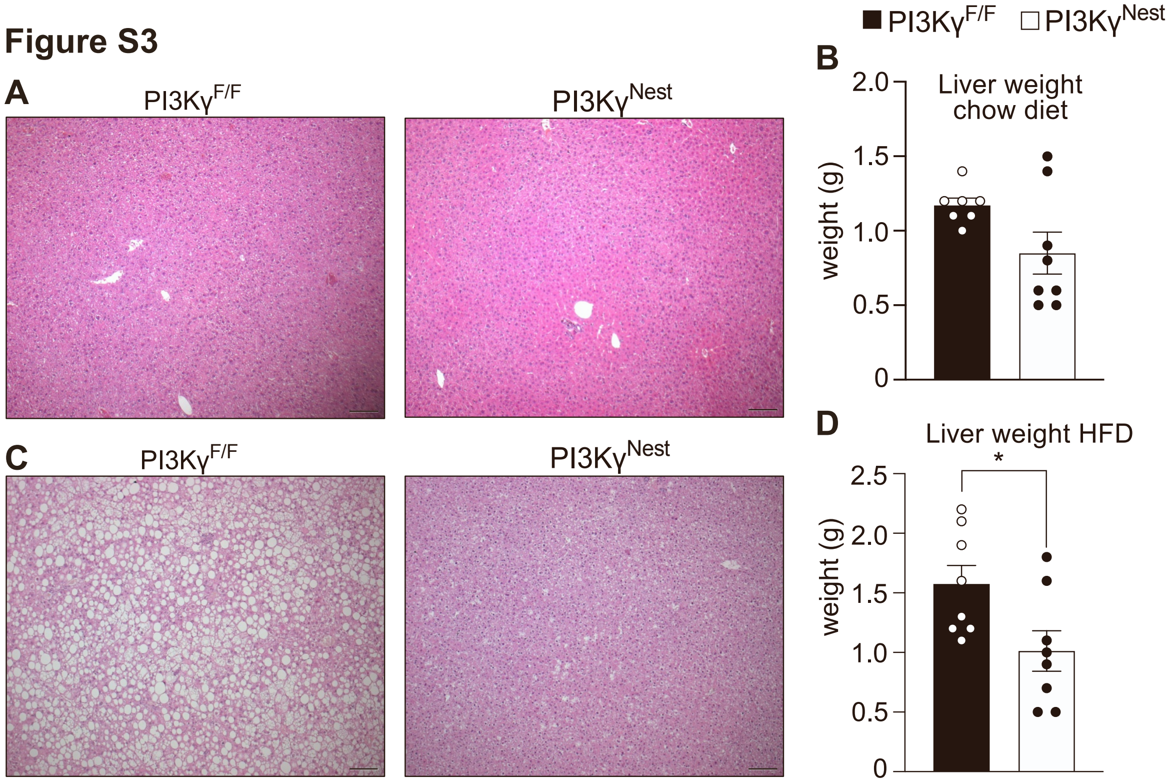

Figure S3. PI3Kγ^{Nest} mice are protected from HFD-induced steatosis. Related to Figure 3.

(A) Representative images of liver histology of the chow-fed PI3K$\gamma^{F/F}$ and PI3Kγ^{Nest} mice from Figure S2.

(B) Liver weight for the mice in A.

(C) Representative images of liver histology of HFD-fed PI3K$\gamma^{F/F}$ and PI3Kγ^{Nest} mice from Figure S2.

(D) Liver weight for the mice in C.

n = 7-8 mice for A and B, n = 8 for C and D. Scale Bar 100 μm.

Data are represented as mean ± SEM. Statistical analysis was performed using Mann-Whitney analysis.

Figure S4

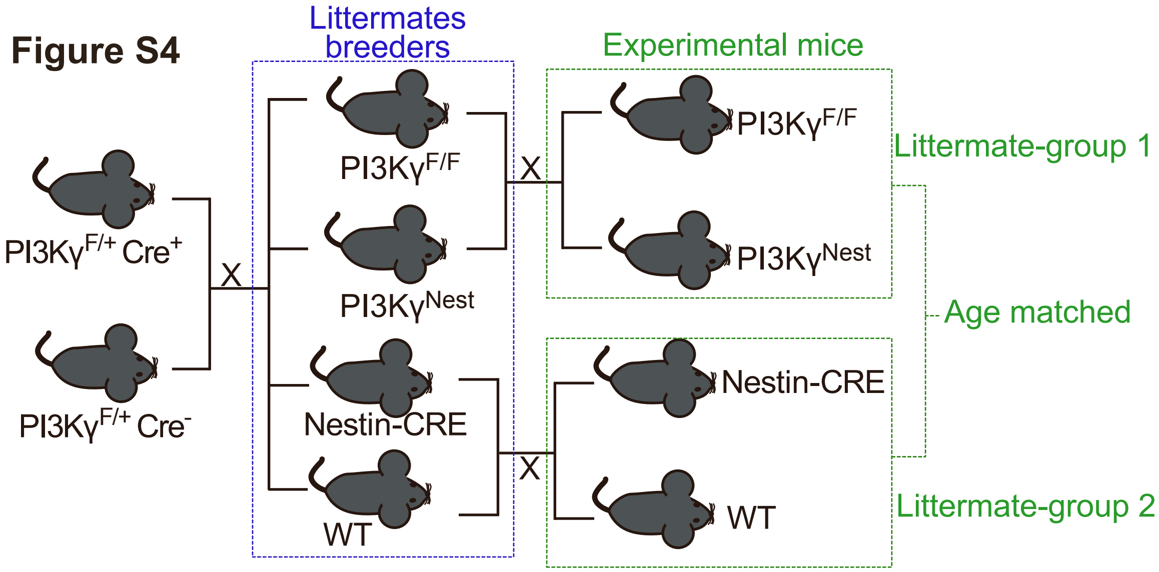

Figure S4. Breeding scheme for experimental mice with a control group on the metabolic effects on neuronal cre expression. Related to Figure

Graphical representation of the breeding scheme used to obtain PI3Kγ^{Nest} and littermate control PI3Kγ$^{F/F}$ mice, and Nestin-CRE and littermate control WT mice. Mice heterozygote for PIK3CG LoxP floxed E3 and E4 Nestin-Cre positive (PI3Kγ$^{F/+}$ Cre^{+}) were crossed with PIK3CG LoxP floxed heterozygote Nestin-Cre negative mice (PI3Kγ$^{F/+}$ Cre^{+}). Littermate PI3Kγ$^{F/F}$, PI3Kγ^{Nest}, WT, and Nestin-CRE mice were divided into two parallel and synchronized breeding groups PI3Kγ$^{F/F}$ crossed with PI3Kγ^{Nest}; and littermate WT mice crossed with Nestin-CRE mice (Littermate breeders). This breeding generated two age-matched littermate groups (Experimental mice).

Figure S5

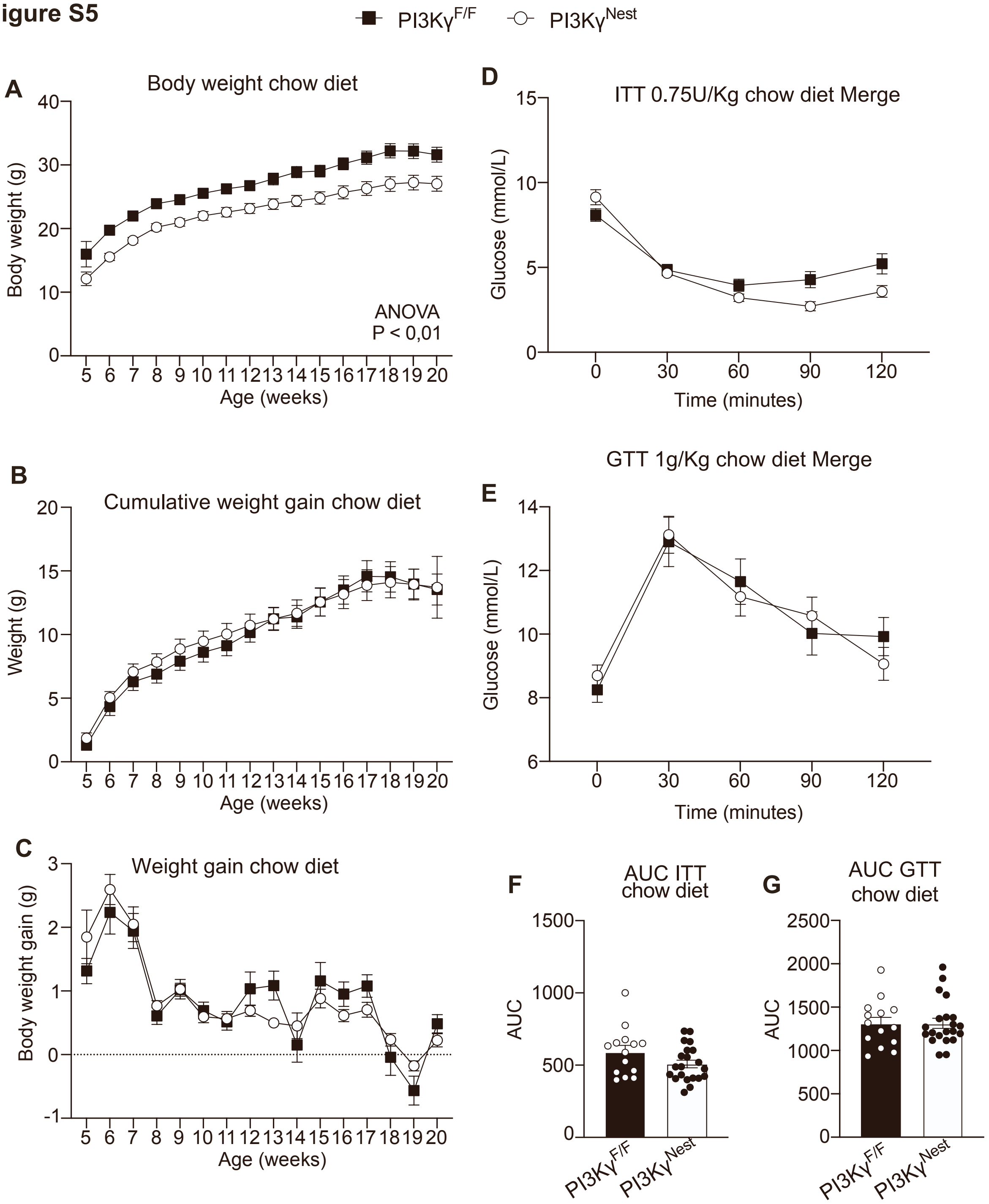

Figure S5. Figure S3. The phenotype of PI3Kγ^{Nest} mice on chow diet in all cohorts combined.

(A) Body weight growth curves of PI3Kγ$^{F/F}$ and PI3Kγ^{Nest} mice kept on a chow diet.

(B) Cumulative weight gain of PI3Kγ$^{F/F}$ and PI3Kγ^{Nest} mice kept on a chow diet.

(C) Weekly weight gain of PI3Kγ$^{F/F}$ and PI3Kγ^{Nest} mice kept on a chow diet.

(D) Insulin tolerance test of PI3Kγ$^{F/F}$ and PI3Kγ^{Nest} mice kept on a chow diet.

(E) Glucose tolerance test of PI3Kγ$^{F/F}$ and PI3Kγ^{Nest} mice kept on a chow diet.

(F) Area under the curve of the ITT in D.

(H) Area under the curve of the GTT in E.

n=14-21.

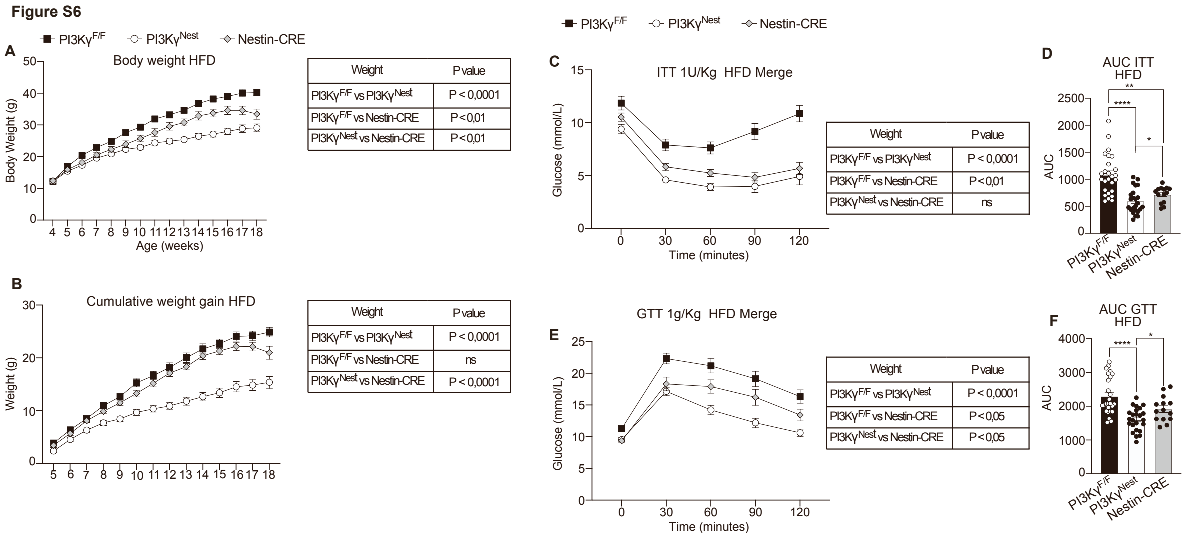

Figure S6. The phenotype of PI3Kγ^{Nest} mice on HFD in all cohorts combined.

(A) Body weight growth curves of PI3Kγ$^{F/F}$,Nestin-CRE, and PI3Kγ^{Nest} mice kept on HFD.

(B) Cumulative weight gain of PI3Kγ$^{F/F}$ and PI3Kγ^{Nest} mice kept on a chow diet.

(C) Insulin tolerance test of PI3Kγ$^{F/F}$ and PI3Kγ^{Nest} mice kept on HFD.

(D) Area under the curve of the ITT in C.

(E) Glucose tolerance test of PI3Kγ$^{F/F}$ and PI3Kγ^{Nest} mice kept on HFD

(F) Area under the curve of the GTT in E.

n=14-27 for A, B, and n=14-26 for C-F.

Figure S7

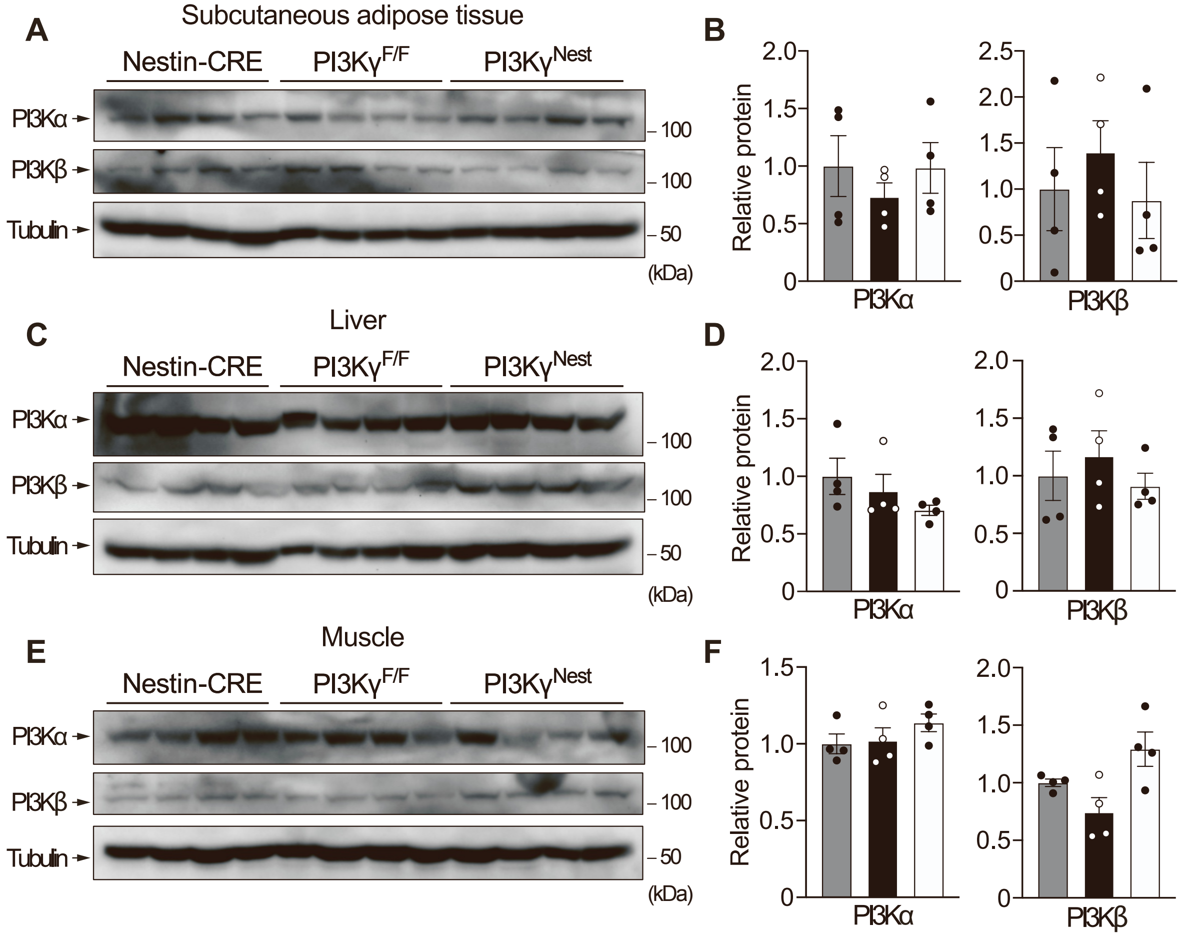

Figure S7. PI3Kα and PIK3β abundances in WAT, Liver, and Muscle.

(A) Immunoblot analysis of PI3Kα and PIK3β protein abundances in WAT.

(B) Quantification of the immunoblots in A.

(C) Immunoblot analysis of PI3Kα and PIK3β protein abundances in Liver.

(D) Quantifications of the immunoblots in C.

(E) Immunoblot analysis of PI3Kα and PIK3β protein abundances in muscle.

(F) Quantifications of the immunoblots in E.

n=4.
